# Durvalumab-induced thyroiditis in a patient with non-small cell lung carcinoma: a case report and review of pathogenic mechanisms

**DOI:** 10.1186/s12902-022-01190-5

**Published:** 2022-11-22

**Authors:** Jeroen M. K. de Filette, Stéphanie André, Lynn De Mey, Sandrine Aspeslagh, Rafik Karmali, Bart J Van der Auwera, Bert Bravenboer

**Affiliations:** 1 Department of Endocrinology, Brugmann University Hospital, Université Libre de Bruxelles, Brussels, Belgium; 2 Department of Endocrinology, UZ Brussel, Laarbeeklaan 101, 1090 Brussels, Belgium; 3 Department of Pulmonary Medicine, Saint-Pierre University Hospital, Rue Haute 322, 1000 Brussels, Belgium; 4 Université Libre de Bruxelles, Brussels, Belgium; 5 Department of Pulmonary Medicine, Brugmann University Hospital, Brussels, Belgium; 6 Department of Nuclear Medicine, UZ Brussel, Laarbeeklaan 101, 1090 Brussels, Belgium; 7 Department of Medical Oncology, Vrije Universiteit Brussel (VUB), Universitair Ziekenhuis Brussel (UZ Brussel), Laarbeeklaan 101, 1090 Brussels, Belgium; 8 Diabetes Research Center, Vrije Universiteit Brussel, Brussels, Belgium

**Keywords:** Durvalumab, Thyroiditis, Immune Checkpoint Inhibitors, HLA, Case Report

## Abstract

**Background:**

Immune checkpoint inhibitors (ICI) targeting cytotoxic T-lymphocyte-associated protein 4 (CTLA-4), programmed cell death protein 1 and its ligand (PD-1/PD-L1) have become the current standard-of-care for advanced cancers. This novel therapeutic approach comes with its costs in the form of immune-related adverse events (irAE), including endocrinopathy.

**Case presentation:**

A 63-year-old woman was diagnosed with a non-small cell lung carcinoma of the right superior lobe, cT3N2M0. She developed thyrotoxicosis followed by hypothyroidism induced by consolidation immunotherapy with durvalumab (anti-PD-L1). Analysis of the human leukocyte antigen (HLA) region showed HLA-DR4 (susceptible) and DR13 (protective). The possible mechanisms are subsequently discussed in detail.

**Conclusions:**

The case of a patient with thyroiditis associated with the PD-L1 inhibitor durvalumab is described, highlighting the need for proactive monitoring of thyroid hormone levels. Identifying biomarkers associated with an increased risk of ICI-induced side effects (such as HLA) is of interest for better patient selection, optimal management and improved understanding of the mechanisms involved.

## Background

Immune checkpoint inhibitors (ICI) targeting cytotoxic T-lymphocyte-associated protein 4 (CTLA-4), programmed cell death protein 1 and its ligand (PD-1/PD-L1) have become the current standard-of-care for many advanced cancers [1]. However, this novel therapeutic approach comes with its costs in the form of immune-related adverse events (irAE). Endocrinopathy is a well-recognized side effect by now, with a distinct role for CTLA-4 blockade (with ipilimumab) in hypophysitis and PD-1/PD-L1 blockade in thyroid dysfunction [2]. The PD-1 inhibitors nivolumab and pembrolizumab were FDA-approved in 2014, while the PD-L1 inhibitors followed shortly thereafter (avelumab, 2015; atezolizumab, 2016; durvalumab, 2017; cemiplimab, 2018) [3]. As such, the thyroid dysfunction has been more comprehensively described with PD-1 inhibitors than with PD-L1 inhibitors due to longer clinical experience [2, 4]. In this report, a patient with thyrotoxicosis followed by hypothyroidism associated with the PD-L1 inhibitor durvalumab is described, including analysis of the human leukocyte antigen (HLA) region. The possible mechanisms involved are subsequently discussed in detail.

## Case presentation

**Fig. 1:**
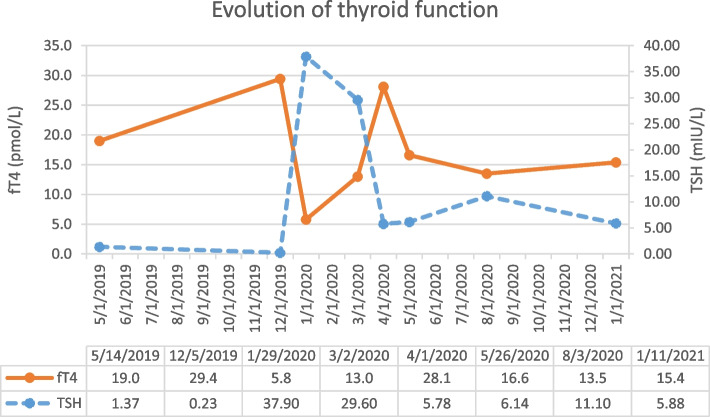
Evolution of thyroid function during durvalumab-associated thyroiditis. Mild thyrotoxicosis evolved into hypothyroidism requiring levothyroxine replacement therapy. The thyroid function tests eventually evolved favourably after insistence on adequate therapeutic compliance

A 63-year-old woman was diagnosed in May 2019 with a non-small cell lung carcinoma of the right superior lobe, cT3N2M0, stage IIIB, after routine chest imaging performed for chronic smoking. She was known with liver cirrhosis Child B-C caused by excessive alcohol intake and allergy to iodinated contrast agents. There was no personal or family history of endocrinopathy. Histology of the affected lymph nodes showed negative NGS, ALK and ROS-1 status; the PD-L1 expression was > 50%. The patient was initiated on two cycles of radio-chemotherapy with carboplatin and vinorelbine. She showed a partial therapeutic response on ^18^F-FDG PET-CT imaging with the disappearance of the hypermetabolism in the paratracheal lymph node, but persistence of mild hypermetabolism in the pulmonary nodules. In October 2019, the patient was started on consolidation immunotherapy with durvalumab (anti-PD-L1) at a dose of 10 mg/kg every two weeks, with the intended treatment duration of one year. A routine blood sampling performed before the first chemotherapy (May 2019) showed normal thyroid function with a TSH of 1.37 mU/L (reference range: 0.27–4.20) and free T4 of 19.0 pmol/L (reference range: 12.0–22.0). The thyroid function was not rechecked before the first durvalumab dosing. In December 2019, after three cycles, the patient presented herself at the pneumology department with fatigue, weight loss (2 kg in two months) and increased shortness of breath. She was afebrile and normotensive. Laboratory analysis showed a decreased TSH (0.23 mU/L) and increased free T4 (29.4 pmol/L). Mild hyperthyroidism was suspected and the immunotherapy was continued as planned. Approximately 6 weeks later, the thyroid function evolved into hypothyroidism (with TSH 37.90 mU/L; free T4 5.8 pmol/L) and treatment with levothyroxine 50 mcg daily was started. Initially, there was a lack of improvement due to limited therapeutic compliance, but the thyroid function eventually evolved favourably, as shown in Fig. 1. Thyroperoxidase antibodies (TPO, at the time of hypothyroidism) were positive (216 kUI/L, reference range: < 34), while the TSH receptor antibodies (TRAb, analysis of stimulating or blocking) were negative. Ultrasound examination of the thyroid was normal, demonstrating a homogeneous, isoechogenic gland with normal vascularisation. Thyroid scintigraphy was not performed. Analysis of the immunogenetic background was performed by HLA typing. This resulted in the A-B-C haplotypes: A*02, -; B*15,*40; C*03,*07; the DR haplotypes: DRB1*04,*13, and the DQ haplotypes: DQB1*03,*06. Durvalumab was administered from October 2019 to June 2020, when she developed ICI-associated pneumonitis that resolved after stopping the immunotherapy. The patient developed no other irAE.

## Discussion and conclusions

The case of a patient with thyroiditis associated with the PD-L1 inhibitor durvalumab is described. Thyroid dysfunction with ICI occurs primarily with PD-1/PD-L1 blockade [2, 4]. Classically, the pattern of an inflammatory, destructive thyroiditis is observed, with or without initial thyrotoxicosis, which evolves into hypothyroidism. Increased uptake of ^18^FDG compatible with inflammatory thyroiditis has been described previously [5–7]. The full spectrum of thyroid dysfunction associated with ICI includes Graves’ disease, and several cases have been reported with PD-1 blockade (nivolumab or pembrolizumab) [8–12]. In two of these cases, the diagnosis was confirmed on radioisotope scintigraphy while TRAb were negative [8, 9]. Graves’ ophthalmopathy was also reported with ipilimumab [13, 14], nivolumab [15], and a combination of CTLA-4 and PD-L1 blockade (with tremelimumab and durvalumab) [16]. Screening for endocrine irAE (including thyroid dysfunction) should be performed at each course of treatment for 6 months, every 2 courses for the following 6 months, and then in case of clinical signs, according to the French endocrine society guidance [17].

One of the limitations of this case report is that another cause of thyroid dysfunction cannot be formally excluded. As evaluation with thyroid scintigraphy is absent, the possibility of TRAb-negative Graves' disease cannot be excluded. However, destructive thyroiditis occurs much more frequently than Graves’ disease with ICI therapy, and the hyperthyroid phase was self-limiting. Furthermore, the patient did not receive iodinated contrast due to a known allergy, and her performance status was satisfactory. Obtaining thyroid tissue would allow additional analysis, but was not performed because of her favorable clinical evolution. High-resolution HLA typing was not performed, missing out on more subtle information. While a single case report is insufficient to draw conclusions, the combination of various reports could contribute to a better understanding of thyroiditis related to ICI therapy, and be an incentive for further prospective study. The ethnic and regional context (iodine supply) must be considered for an accurate interpretation of thyroid dysfunction (with ICI therapy).

This case report illustrates that more attention should be given to the immunogenetic background of oncology patients treated with immunotherapy [18]. 'Conventional' autoimmune thyroid disease (AITD) is a complex subject in terms of immunogenetics. For the non-HLA polymorphisms, there is a common pathway for Hashimoto thyroiditis and Graves' disease, both being associated with specific alleles of CTLA-4 and PTPN22, or TSHR polymorphisms [19]. There also seems to be a causal relationship with HLA polymorphisms. For Hashimoto thyroiditis, the HLA-DR4 subtype is susceptible, while the DR7 and DR13 subtypes are protective [20], based on the study by *Zeitlin *et al. on well-characterized cases of AITD. Our patient has a mixed HLA susceptibility, with the presence of both HLA-DR4 (susceptible) and DR13 (protective). The HLA-DR3 variant is known to increase susceptibility for Graves' disease [21, 22]. The HLA polymorphisms could at least partially explain why variation in side effects occur in patients treated with ICI.

A variety of HLA associations have been described in patients with irAE: for example pruritus (DRB1*11:01) [23], colitis (DQB1*03:01) [23], inflammatory arthritis (DRB1*04:05) [24] and immune-related pneumonitis (B*35 and DRB1*11) [25]. Concerning the endocrinopathy, the association with HLA-DR4 has been observed for diabetes mellitus [26, 27], with further differences in genotype depending on the background of the specific population (DR4-DQ4 and DR9-DQ9 in Asian origin) [27]. Serotypes associated with lymphocytic hypophysitis (HLA-A2, A24, B7, DR1 and DR4) were found in two cases of Japanese origin with nivolumab-induced hypophysitis [28]. In another Japanese patient with isolated adrenocorticotropin deficiency caused by nivolumab-induced hypophysitis, the DRB1*04:05-DQA1*03:03-DQB1*04:01 haplotype was found, which is associated with susceptibility to autoimmune polyglandular syndrome with a pituitary disorder in a Japanese population [29]. The literature is less clear on this subject regarding thyroid dysfunction. In a Japanese population, an association was demonstrated in two cases of Graves' disease (DRB1*04:05 and DPB1*05:01) [11, 12] and in thyroiditis (permanent, B*46:01, DPA1*01:03-DPB1*02:01; temporary, C*14:02; protective, DPA1*02:02-DPB1*05:01) [30].

Furthermore, the role of pre-existing AITD is still incompletely understood in the pathophysiology of thyroid dysfunction with immune therapy. In 'conventional' AITD, the presence of antithyroid antibodies correlates with T-cell infiltration into the thyroid [31]. In the context of ICI, the presence of antithyroid autoantibodies *at baseline* is associated with a higher chance of thyroid dysfunction by some authors [32–34]. In particular, *Muir *et al. found that all 27 patients with positive TPOAb, and 41/42 patients with positive TgAb at baseline, developed thyroid irAE [35]. In a prospective study by Okada et al., the incidence of thyroid dysfunction induced by anti-PD-1 antibodies was markedly higher in patients with anti-thyroid antibody positivity (34.1%, 15/44, vs 2.4%, 4/165) [36]. *Osorio *et al*.* describe that their onset coincided with the development of thyroid dysfunction (7/11 anti-thyroid antibodies developed during pembrolizumab treatment, of which 6/7 with onset of transient hyperthyroidism) [37]. According to yet other authors, thyroid autoantibodies are absent in patients with thyroid dysfunction [38, 39]. It remains to be determined whether the pre-existence of subclinical thyroid autoimmunity can be reliably detected through thyroid autoantibodies *at baseline* and subsequently confers a risk for developing thyroid dysfunction in this population. We hypothesize a primarily T-cell mediated toxicity with a role as a secondary phenomenon for thyroid autoantibodies. An alternative way to study the role of the humoral pathway would be to evaluate ICI in different types of B-cell deficiency. For example, it was demonstrated in murine studies (with B-cell depleting antibodies or strains lacking mature B-cells) that the presence of B-cells is not required for a tumour response to anti-PD-1 therapy [40]. In a patient with hypogammaglobulinemia after rituximab treatment (for marginal zone B-cell lymphoma), a partial response of the PD-1 inhibitor sintilimab was observed in advanced lung adenocarcinoma (co-existing in the same patient) [41]. It would be of interest to more closely examine the effects of ICI therapy in patients with B-cell deficiencies, both in terms of tumour efficacy as well as side effects.

Histological correlates are rare due to the self-limiting nature of this condition, although a few case reports have implied CD8^+^ T cell-mediated destruction of thyroid follicles [42–45]. In one case, morphologic features included the formation of non-necrotizing colloid granulomas [42]; in another, cytopathology included clusters of necrotic cells, lymphocytes and CD163-positive histiocytes [43]. The expression of PD-L1 and PD-L2 has been demonstrated in normal thyroid tissue, suggesting a distinct immunomodulatory role of this checkpoint in the thyroid gland even without pre-existing thyroid autoimmunity [46]. Of further interest is the possibility of increased ^18^FDG uptake in the thyroid gland [5–7], which should be sought on imaging, especially in the first few months after initiating ICI. Lastly, the association between the development of endocrine-related side effects and the oncological response remains controversial. According to the retrospective study by *von Itzstein *et al*.,* patients who develop an abnormal TSH after ICI initiation have a favorable prognosis, while those with an abnormal TSH at baseline have inferior survival [34]. The development of thyroid dysfunction after ICI initiation could reflect the activation of an anticancer immune response, also resulting in autoimmunity against the thyroid gland.

In summary, the case of a patient with thyroiditis associated with the PD-L1 inhibitor durvalumab is reported, highlighting the need for proactive monitoring of thyroid hormone levels. Identifying biomarkers associated with an increased risk of ICI-induced side effects (such as HLA) is of interest for better patient selection, optimal management and improved understanding of the mechanisms involved.

## Data Availability

The datasets used and/or analysed during the current study are available from the corresponding author on reasonable request.

## Abbreviations

AITD: Autoimmune thyroid disease
CTLA-4: Cytotoxic T-lymphocyte-associated protein 4
HLA: Human leukocyte antigen
ICI: Immune checkpoint inhibitors
irAE: Immune-related adverse events
PD-1/PD-L1: Programmed cell death protein 1 / ligand
TPO Ab: Thyroperoxidase antibody
TRAb: TSH receptor antibody
